# Research on Diagnosis Prediction of Traditional Chinese Medicine Diseases Based on Improved Bayesian Combination Model

**DOI:** 10.1155/2021/5513748

**Published:** 2021-06-10

**Authors:** Zhulv Zhang, Jinghua Li, Wanting Zheng, Shaolei Tian, Yang Wu, Qi Yu, Ling Zhu

**Affiliations:** Institute of Information on Traditional Chinese Medicine, China Academy of Chinese Medical Sciences, Beijing, China

## Abstract

Traditional Chinese Medicine (TCM) clinical intelligent decision-making assistance has been a research hotspot in recent years. However, the recommendations of TCM disease diagnosis based on the current symptoms are difficult to achieve a good accuracy rate because of the ambiguity of the names of TCM diseases. The medical record data downloaded from ancient and modern medical records cloud platform developed by the Institute of Medical Information on TCM of the Chinese Academy of Chinese Medical Sciences (CACMC) and the practice guidelines data in the TCM clinical decision supporting system were utilized as the corpus. Based on the empirical analysis, a variety of improved Naïve Bayes algorithms are presented. The research findings show that the Naïve Bayes algorithm with main symptom weighted and equal probability has achieved better results, with an accuracy rate of 84.2%, which is 15.2% higher than the 69% of the classic Naïve Bayes algorithm (without prior probability). The performance of the Naïve Bayes classifier is greatly improved, and it has certain clinical practicability. The model is currently available at http://tcmcdsmvc.yiankb.com/.

## 1. Introduction

The disease diagnosis in TCM has a long history. There are more than 100 disease names recorded in the “Huangdi Neijing,” and 13 formulas are specially designed for diseases [1]. It can be seen that the field of TCM pays great attention to disease diagnosis. “Disease” in TCM is a generalization of basic regularities and contradictions in the entire evolution of the disease, including certain specific symptoms and corresponding syndromes [2]. TCM disease diagnosis refers to the complex process of physicians using various methods, such as inspection, listening, and smelling examination, inquiry, and palpation, to collect patient clinical information and analyze the patient's clinical information based on the theoretical knowledge of TCM and finally confirm the patient's complicated disease. Disease diagnosis is a key link for physicians to diagnosis and treatment of diseases, and its accuracy is directly related to the effect and standardization of clinical diagnosis and treatment. In this study, TCM disease prediction is modelled as a text classification task in natural language processing, which is known to be a domain with high-dimensional feature space challenge [3].

In recent years, deep learning is a focused research direction of machine learning, which seeks to identify a classification scheme with higher predictive performance based on multiple layers of nonlinear information processing. Despite many researches in the field of sentiment analysis [4], topic identification, and genre classification, [5–8] have shown deep learning and ensemble learning, such as recurrent neural network in conjunction with GloVe or attention mechanism, in which the accuracy is superior to conventional supervised learning methods, but, because of the particularity of Chinese medicine field, a large amount of real clinical record is very difficult to collect. Furthermore, conventional supervised learning has better interpretability than deep learning. Therefore, Naïve Bayes is chosen as the research method in this study. In disease diagnosis, the use of mathematical algorithm models can often achieve good results [9]. The Bayesian classification algorithm is a typical statistical method that can be used for reasoning and forecasting research, which was proposed by the British mathematician Thomas Bayes in the 18th century based on the “inverse probabilities” problem. It is based on the Bayesian formula. The method of probabilistic reasoning is utilized to calculate the probability that the sample belongs to a particular class; it assumes that all feature variables *Xk* are independent of each other. This assumption seems a bit unreasonable, but it has been proved by many studies to have better performance in classification tasks [10], which can effectively solve the problem of uncertain knowledge reasoning [11]. Bayesian classification algorithm is widely used in biology [12], transportation [13], meteorology [14], economy [15], medicine [16], and other fields because of its high practicability.

In 1980, a scientific researcher [17] put forward the idea of applying Bayesian algorithm to disease diagnosis of TCM. Qin [18] improved the traditional Naïve Bayesian classification method and applied it to the diagnosis of asthma in TCM and achieved good experimental results. Du [19] applied the improved weighted hidden Naïve Bayes classification algorithm to the actual infertility diagnosis of TCM providing a good idea and method for the modelling of infertility TCM diagnosis. In addition, there are still many related works that have achieved outstanding results [20–23]. The above work has accelerated the pace of diagnostic research in TCM, improved the accuracy, speed, and efficiency of clinical disease diagnosis, and laid a good foundation for artificial intelligence research in TCM. However, due to the limitations of data quality, terminology standard, computing power, and so forth, the TCM disease diagnosis model based on Bayesian algorithm still has certain shortcomings. It needs to be further upgraded and improved to meet the increasing TCM clinical and scientific research needs.

The Big Health TCM Intelligent R&D Center of the Institute of Information, CACMC, has more than ten years of research foundation in TCM informatization, software development, TCM algorithm research, ontology constructing, and TCM data. Based on the research of the center, this research has made certain explorations in the diagnosis and prediction of TCM diseases based on the modified Bayesian joint model. It is introduced as follows.

## 2. Basic Data Preparation

Due to the complexity of TCM diseases, the medical records of some diseases are too scarce, and the guidelines are missing, which leads to serious imbalances in data and affects the effect of machine learning. Therefore, this study is based on the top 100 common diseases in Dongzhimen Hospital of Beijing University of TCM (see Table 1). The data of the study mainly comes from the medical record data of the ancient and modern medical record cloud platform (http://www.yiankb.com/totaldatavolumeof300,000+), as well as the practical clinical guidelines of the TCM clinical decision support system (https://www.tcmcds.com/totaldatavolume4000+), developed by the Institute of Information, CACMS, extracts the medical records and guidelines data of 100 common diseases in Table 1, and removes the data of multidisease diagnosis. There are a total of 37103 items, of which 2/3 are the training data, and 1/3 are the test data.

## 3. Data Cleaning

It is well acknowledged that the problem of data cleaning is the basic work in machine learning and deep learning. In this study, ontology data (Table 2) in more than 80,000 fields of TCM diseases, symptoms, and signs in the background of the TCM clinical auxiliary decision support system are used as the data standard, and the TCM disease diagnosis data and symptom data in the medical records and guide data are standardized; for example, “Menstrual period” is standardized as “late menstrual period,” and “Easy to wake up early,” “Wake up midnight,” “Wake up frequently every night,” “Difficulty falling asleep,” and other specifications are “Insomnia.” The standard of symptoms and TCM disease names is an aid to TCM diseases intelligent diagnosis which is very important. Because the Bayesian-based TCM disease diagnosis prediction model does not check the established symptom words but supports the doctor to input the symptom words in natural language, the recognition of the symptom words and the matching rate in the existing corpus have a large impact on the accuracy of decision-making.

Based on the characteristics of the description of symptoms in the medical record corpus, abandoning the traditional-dictionary-based and statistical and machine-learning-based word segmentation methods, the medical record corpus is segmented using a comma as a segmentation method.

## 4. Method

This project uses the Naïve Bayes method for modelling. Naïve Bayes is a simplification of the Bayesian method. It is based on the conditional independence between each feature and the label. The joint probability of characteristics and the label need to be obtained in the Bayesian method.

For a sample *D* to be classified, its sample attribute *X* = {*X*1, *X*2, ..., *Xn*} and categorical variable *C* = {*C*1, *C*2, ..., *Cm*}; according to Bayes' theorem, the posterior probability can be represented by the prior probability *P* (*C*), the class conditional probability *P* (*X*|*C*), and the standardized constant *P* (*X*).

(1) $$\begin{array}{c} P\left(C|X\right)=\frac{P\left(X|C\right)P\left(C\right)}{P\left(X\right)}. \end{array}$$

While NB assumes that all feature variables *Xk* are independent of each other, given category C and sample attribute *X*, the conditional independence assumption can be expressed as

(2) $$\begin{array}{c} P\left(X|C=c\right)=\prod_{k=1}^{n}P\left(X_{k}|C=c\right). \end{array}$$

According to the above formula, if you want to calculate the probability *p* (Disease *A*| Symptom *A*, Symptom *B*, Symptom *C*) that Symptom *A*, Symptom *B*, and Symptom *C* are diagnosed as Disease *X*, you need to get *P* (Symptoms) in the data set Symptom *A*, Symptom *B*, Symptom *C*, Disease *X* joint probability; if there is no cooccurrence of Symptoms *A*, *B*, and *C* and a certain disease in the data set, the Bayesian method cannot give a result.

In order to make better use of the excellent performance of Naïve Bayes in classification, while avoiding this kind of nondiagnostic recommendation, and ensuring the accuracy of the classification results, this study uses an improved Naïve Bayes model to calculate the conditional probability; namely, when calculating *p*(Disease*X*)/P(Symptom*A*,  Symptom*B*,  Symptom*C*) you only need to calculate *p*(Symptom*A*)*|*Disease*X*/*P*(Symptom*A*), *p*(Symptom*B|*Disease*X*)/*P*(Symptom*B*), and *p*(Symptom*C|*Disease*X*)/*P*(Symptom*C*) for the case where there is no Disease *X* and Symptom A in the data set, and give *P* (Disease *X*|Symptom *A*) a very small number. See formulas (3) and (4).

The Bayesian formula is as follows:

(3) $$\begin{array}{c} P\left(\text{Disease}X|\text{Symptom}A,\text{ Symptom}B,\text{ Symptom}C\right)=\frac{p\left(\text{Symptom}A,\text{ Symptom }B,\text{ Symptom}C|\text{Disease}X\right)}{P\left(\text{Symptom}A,\text{ Symptom}B,\text{Symptom}C\right)}*P\left(\text{Disease}X\right). \end{array}$$

Naïve Bayes is as follows:

(4) $$\begin{array}{c} \frac{P\left(\text{Symptom}A|\text{Disease}X\right)*P\left(\text{Symptom}B|\text{Disease}X\right)*P\left(\text{Symptom}C\right)|\text{Disease}X}{P\left(\text{Symptom}A\right)*P\left(\text{Symptom}B\right)*P\left(\text{Symptom}C\right)}\;*P\left(\text{Disease}X\right). \end{array}$$

As mentioned earlier, Naïve Bayes requires each feature to be independent of the others, but it is difficult to make all the features independent of each other in the real world; and some studies have shown that Naïve Bayes performs well not only in the classic situation where each feature is independent of the others but also in other situations [24, 25], which also motivates us to develop this research to increase the use of Bayesian scenarios and to find suitable methods for the auxiliary diagnosis of TCM diseases.

As we all know, in the diagnosis of TCM disease, the various symptoms of each disease are related. In order to obtain a better generalization ability of the model, this study uses formula (5) as the calculation method, which may lose a certain accuracy. From formula (4), we can get the following.

Naïve Bayes is as follows:

(5) $$\begin{array}{c} P\left(\text{Disease}X|\text{Symptom}A,\text{ Symptom}B,\text{ Symptom}C\right)=\frac{P\left(\text{Symptom}A|\text{Disease}X\right)}{P\left(\text{Symptom}A\right)}*\frac{P\left(\text{Symptom}B|\text{Disease}X\right)}{P\left(\text{Symptom}B\right)}*\frac{P\left(\text{Symptom}C|\text{Disease}X\right)}{P\left(\text{Symptom}C\right)}. \end{array}$$

Formula (5) is equivalent to formula (4). It can be seen that, after deformation, each (disease, symptom) cooccurrence pair is regarded as a feature item, and each feature item has the same weight.

In the diagnosis and prediction of TCM diseases, there is a situation where a group of immediate symptoms correspond to two disease diagnoses, which belong to two categories. Symptom*A*, Symptom*B*, and Symptom*C* and Disease*X*1 and Disease*X*2 are classified into two categories, and it is equivalent to judge

(6) $$\begin{array}{c} \frac{P\left(\text{Disease}X1|\text{Symptom}A,\text{ Symptom}B,\text{ Symptom }C\right)}{P\left(\text{Disease }X2|\text{Symptom}A,\text{ Symptom}B,\text{ Symptom }C\right)}>1, \end{array}$$

that is, the probability of Disease*X*1 is higher than the probability of Disease*X*2. According to the Naïve Bayes formula, we can get

(7) $$\begin{array}{c} \frac{P\left(\text{Disease }X1|\text{Symptom}A,\text{ Symptom}B,\text{ Symptom}C\right)}{P\left(\text{Disease}X2|\text{Symptom}A,\text{ Symptom}B,\text{ Symptom}C\right)}=\frac{P\left(\text{Symptom}A|\text{Disease}X1\right)*P\left(\text{Symptom}B|\text{Disease}X1\right)*P\left(\text{Symptom}C|DiseaseX1\right)*P\left(\text{Disease}X1\right)}{P\left(\text{Symptom}A|\text{Disease}X2\right)*P\left(\text{Symptom}B|\text{Disease}X1\right)*P\left(\text{Symptom}|\text{Disease}X1\right)*P\left(\text{Disease}X2\right)}. \end{array}$$

Since the division of formula (7) is prone to produce too small numbers, take the log function on both sides to get log

(8) $$\begin{array}{c} \frac{P\left(\text{Disease}X1\right)|\text{Symptom}A,\text{ Symptom}B,\text{ Symptom}C}{P\left(\text{Disease}X2\right)|\text{Symptom}A,\text{ Symptom}B,\text{ Symptom}C}=\log\frac{P\left(\text{Symptom}A|\text{Disease}X1\right)}{P\left(\text{Symptom}A|\text{Disease}X2\right)}+\log\frac{P\left(\text{Symptom}B|\text{Disease}X1\right)}{P\left(\text{Symptom}B|\text{Disease}X2\right)}+\log\frac{P\left(\text{Symptom}C|\text{Disease}X1\right)}{P\left(\text{Symptom}C|\text{Disease}X1\right)}+\log\frac{P\left(\text{Disease}X1\right)}{P\left(\text{Disease}X2\right)}. \end{array}$$

The left side of formula (8)'s equal sign greater than 0 is classified as Disease*X*1, and the classification result can be obtained. The above disease prediction example considers the logistic regression model, which is equivalent to using the prediction result of the linear regression model to approximate the logistic ratio of the posterior probability; then we have the following formula:

(9) $$\begin{array}{c} \log\frac{P\left(\text{Disease}X1\right)|\text{Symptom}A,\text{ Symptom}B,\text{ Symptom}C}{P\left(\text{Disease}X2\right)|\text{Symptom}A,\text{ Symptom}B,\text{ Symptom}C}=w1*\text{Symptom}A+w2*\text{Symptom}B\;+w3*\text{Symptom}C+b. \end{array}$$

*w* is the feature item which means the weight of the symptom in formula (9). If the feature item is binary discrete, the value is [0, 1] in formula (9); then formula (10) can be produced:

(10) $$\begin{array}{c} \log\frac{P\left(\text{Disease}X1|\text{Symptom}A,\text{ Symptom}B,\text{ Symptom}C\right)}{P\left(\text{Disease}X2|\text{Symptom}A,\text{ Symptom}B,\text{ Symptom}C\right)}=w1+w2+w3+b. \end{array}$$

It can be seen that formulas (8) and (10) are very similar. The feature items are added together, and an independent item is added. The log*P*Disease*X*1/*P*Disease*X*2 in formula (8) is similar to *b* in formula (10). The relationship between Naïve Bayes and logistic regression is deduced here. The difference is that each feature item of logistic regression has *W*_1_, *W*_2_,… weights. Naïve Bayes (formula (8)) is here regarded as the equal weight of each feature item, or weight is obtained only by the ratio of the conditional probability of each feature. For example, the weight of the feature item of Symptom *A* is calculated by *P*(Symptom*A|*Disease*X*)/*P*(Symptom*A*), and the log-linear in Naïve Bayes and logistic regression have different effects.

**Figure 1:**
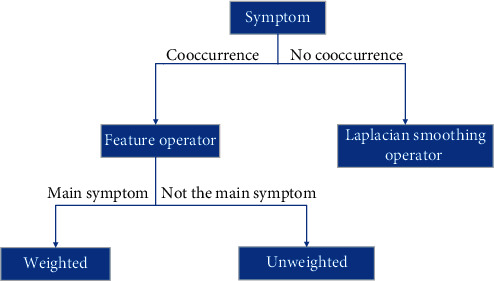
Main symptom weighted diagram.

The data set in this study mainly comes from clinical medical records. According to the experts' experience, the first three symptoms in the clinic are more likely to be the main symptoms and have the largest weight in the diagnosis prediction, that is, the greatest contribution to the diagnosis of the disease. Therefore, this article uses a method to add a weight coefficient greater than 1 to the first three main symptoms in the study. When calculating the feature item operator of each symptom, if the symptom and disease cooccur in the data set, follow formula (5), and if there is no cooccurrence, according to Laplacian smoothing calculation, the feature operator will get a very small value, so that each input symptom feature operator would have a value. If the symptom is the main symptom (the first 3 inputs), add a coefficient greater than 1 in front of the feature item operator to increase the weight of the operator. See Figure 1.

The symptom set {*Xi*} was input to calculate all the diseases {*Yi*} involved in the symptoms, while calculating *P*(*Yi*|*X*1, *X*2...) according to each disease in order to get the result set of the posterior probability of the disease {*P*(*Y*1), *P*(*Y*2), ... *P*(*Yi*)}, the top 3 in the result set as the recommended result.

In this paper, formula (5) is used to calculate the posterior probability of disease. From formula (5), two calculation methods of weighted and unweighted main symptoms are derived through deformation and data processing. Considering the meaning of Bayesian formula, we can understand it from another perspective:

(11) $$\begin{array}{c} P\left(Y|X\right)=\frac{P\left(Y|X\right)}{P\left(X\right)}*P\left(Y\right), \end{array}$$

where *P*(*Y*) term is the prior probability of *Y*, the *P*(*X|Y*)/*P*(*X*) term is regarded as a feature term operator called likelihood, the conditional probability of numerator *y* to *x*, numerator *p*(*x*) is the normalization term, and *P*(*Y*|*X*) on the left side of the equation is the posterior probability of *Y* under the fact that *x* occurs; then the probability of *Y* occurring after *X* has changed from *p*(*y*) to *p*(*y*|*x*), and the original probability of *P*(*Y*) is the prior probability of a disease in the data set in this study. Both sides of formula (11) are divided by *p*(*y*) to get

(12) $$\begin{array}{c} \frac{P\left(y|x\right)}{P\left(y\right)}=\frac{P\left(X|Y\right)}{P\left(X\right)}. \end{array}$$

**Figure 2:**
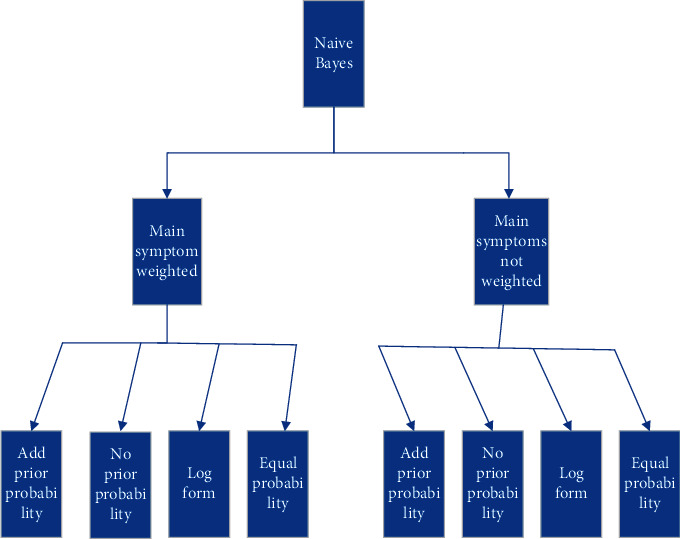
Eight different Bayesian algorithms.

**Figure 3:**
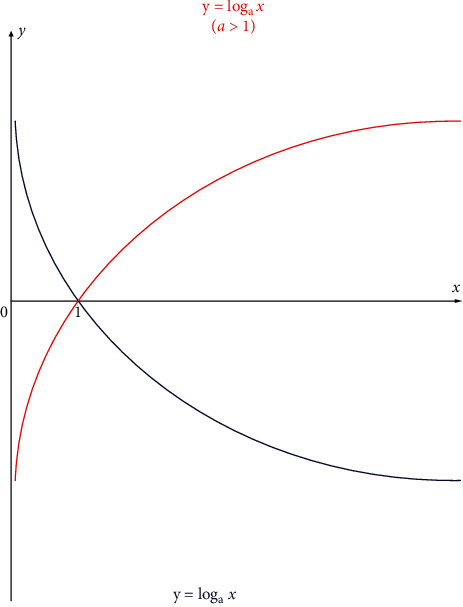
Log form.

The left side of formula (12) can be regarded as the rate of change between the posterior probability of *Y*(*p*(*y*/*x*)) and the prior probability which also cleverly avoids the problem of imbalance in the prior probability of *p*(*y*) in the data set. Therefore, we have made a modification and update for the Naïve Bayes formula, which are the method of adding prior probability and the method of not adding prior probability will be discussed later. The above is the first algorithm used in this article. All eight different Bayesian algorithms used in this article can be shown in Figure 2. In addition, log form is shown in Figure 3.

We have transformed formula (5) into formula (12) in the previous article. Formulas (8) and (9) are logarithmic forms of Naïve Bayes and logistic regression, respectively. The linear functions of the two formulas are different. The basic assumption of Naïve Bayes is that each dimension of the sample is conditionally independent; that is, *P*(*X*1, *X*2, *X*3..) = *P*(*X*1) ∗ *P*(*X*2) ∗ *P*(*X*3)...; in order to avoid underflow of floating-point numbers, we add a log function in front to get formula (8), which does not change the monotonicity. It can be seen that when the log base is bigger than log(*P*(*y*)), which is the prior probability, it becomes smoothed under the action of the log function. Furthermore, this term is changed from multiplication to addition, which reduces the influence of the prior probability to a certain extent. For example, the number of a certain disease in the data set is small; that is, the priori probability product term is very small, resulting in a very small posteriori value, so the algorithm adds a branch of log form.

In order to solve the problem of imbalanced prior probability, we also adopted an oversampling method to make 100 diseases in the data set to be processed with equal probability. Here we assume that the prior probability of each disease is 1/100 and then use the main symptoms weighted and unweighted methods for calculation.

## 5. Results and Discussion

In the experiment, we use 8 calculation methods of Naïve Bayes method and its variants shown in Figure 2, using 3-fold cross-validation of the data. We get a list of the diseases involved in all symptoms in each piece of test data. According to the 8 algorithms, we get the ranking of the disease probabilities. The diseases with the top 3 probabilities are used as the recommended results. In the evaluation of the results, if the recommended results hit the disease corresponding to the data then it is recorded as the correct prediction, according to this rule to calculate the accuracy rate, shown in Table 3.

## 6. Conclusion

As can be seen from the above figure, this study is based on the classic TCM syndrome differentiation idea and proposes an algorithm improvement method for the weighting of the main symptoms. Among all 8 modified Naïve Bayes algorithms, the algorithm with the highest accuracy is the weighted and equal probability algorithm for the main symptoms, reaching 84.2% of accuracy, which is 15.2% higher than the 69% of the classic Naïve Bayes algorithm (without prior probability), which greatly improves the performance of the Naïve Bayes classifier and has certain clinical practicability. The model is currently available at http://tcmcdsmvc.yiankb.com/.

However, due to the privacy of TCM medical record corpus, it is difficult to obtain large-scale, real, effective, and high-quality medical record corpus. Moreover, the diagnosis of TCM disease is vague, and the boundary between disease and symptoms is not very clear. For example, cough is also the name of the disease and the name of the syndrome, which makes it difficult to improve the accuracy of the prediction and recommendation of TCM disease diagnosis. There is also some room for improvement in the process of this research. For example, word segmentation is too granular according to punctuation. The matching between user input symptoms and Bayesian corpus symptoms should be too dependent on the domain ontology, and if the ontology is not covered, its accuracy will be greatly reduced. Both issues need optimization in the next version.

Secondly, the main symptoms weight coefficient is artificially set, with a certain degree of randomness and uncontrollability. In the future, on the basis of having more labeled corpus, we can further try more updated algorithms to provide methodological guarantee for optimizing the performance of the TCM clinical decision-making system. Furthermore, some schemes based on conventional machine learning method and ensemble learning methods (such as Boosting, Bagging, and Random Subspace) have achieved good performance in text genre classification and sentiment analysis [26–28], which shall be a promising method that can be explored in subsequent studies [29]. Meanwhile, some data mining method and feature selection methods [30, 31] can be useful to discover the relationship between disease and symptoms and improve the accuracy of TCM disease diagnosis recommendation. Further research may yield more promising results by exploring more methods in this study.

## Figures and Tables

**Table 1:** Top 100 common diseases in TCM.

Top100 common diseases			
Cough	Postpartum depression	Heat stranguria	Summer nonacclimation
Insomnia	Amenorrhea	Spontaneous sweating	Enterobiasis
Menstrual disorder	Pelvic mass in woman	Night sweating	Dysphagia
Common cold	Metrorrhagia	Asthma syndrome	Stranguria due to hematuria
Constipation	Leukorrheal diseases	Prospermia	Qi goiter
Lumbodynia	Advanced menstruation	Impotence	Regurgitation
Headache	Menorrhagia	Postpartum hypogalactia	Somnolence
Consumptive disease	Delayed menstruation	Acute mastitis	Consumptive thirst involving kidney
Chest discomfort	Lump in breast	Acute appendicitis	Dementia
Palpitation	Menostaxis	Enuresis	Hemoptysis
Stomach ache	Apoplexy	Eczema	Mumps
Stomach distension	Consumptive thirst	Nodule in breast	Hysteria
Arthralgia syndrome	Vomiting	Anorexia	Heat stroke and sunstroke
Tinnitus	Oral aphthae in children	Epistaxis	Epilepsy
Abdominal pain	Gastric discomfort	Purpura	Lung distention
Bone bidisease	Acute and chronic sinusitis	Jaundice	Lung abscess
Depression syndrome	Globus hystericus	Tympanites	Gall
Hypomenorrhea	Diarrhea	Urolithic stranguria	Sallow complexion
Wind-warm disease with lung heat	Hypochondriac pain	Frozen shoulder	Dacryocystitis
Vertigo	Facial palsy	Thrush	Cold tear induced by wind
Fever	Edema	Snake-like sores	Manic-depressive psychosis
Infertility	Aphtha	Deafness	Lung-wind acne
Dysmenorrhea	Frequent micturition	Stiff neck	Hemorrhoidal disease
Menopausal syndrome	Infantile malnutrition	Neck arthralgia	Hidden rashes
Premenstrual syndrome	Irregular menstrual cycle	Stranguria due to overstrain	Dysentery

**Table 2:** Domain ontology status.

Classification	Quantity
Western medicine disease	3041
TCM disease	2212
Syndromes	844
Symptom	69649
Tongue and pulse	8307
	84053

**Table 3:** Accuracy of 8 algorithms.

	Calculation method	Calculation formula	Accuracy (%)
Main symptom weight (for the first 3 symptoms, if the disease has cooccurrence, multiply it by the weight coefficient)	Add prior probability	*P*(Disease*X|*Symptom*A*, Symptom*B*, Symptom*C*)=*P*(Symptom*A|*Disease*X*)/*P*(Symptom*A*)*∗P*(Symptom*B|*Disease*X*)/*P*(Symptom*B*)*∗P*(Symptom*C||*Disease *X*)/*P*(Symptom*C*)*∗P*(Disease*X*) (for the first 3 symptoms, if the disease has cooccurrence, multiply it by the weight coefficient)	73
	No prior probability	*P*(Disease*X|*Symptom*A*, Symptom*B* Symptom*C*)/*P*(Disease*X*)=*P*(Symptom*A|*Disease*X*)/*P*(Disease*A*)*∗P*(Symptom*B|*Disease*X*)/*P*(Symptom*B*)*∗P*(Symptom*C|*Disease*X*)/*P*(Symptom*C*)	69
	Log function form	log(*P*Disease*x*1*|*Symptom*A*, Symptom*B*, Symptom*C*)=log*P*(Symptom*A|*Disease*X*1)/*P*(Symptom*A|*Disease*X*2)+log*P*(Symptom*B|*Disease*X*1)/*P*(Symptom*B|*Disease*X*2)+log*P*(Symptom*C|*Disease*X*1)/*P*(*S*ymptom*C|*Disease*X*2)+log*P*Disease*X*1/*P*Disease*X*2	77
	Equal probability	*P*(Disease*X|*Symptom*A*, Symptom*B*, Symptom*C*=*P*(Symptom*A|*Disease*X*)/*P*(Symptom*A*))*∗P*(Symptom*B|*Disease*X*)/*P*(Symptom*B*)*∗P*(Symptom*C|*Disease*X*)/*P*(Symptom*C*)*∗P*(Disease*X*)	84.2

Main symptoms are not weighted	Add prior probability	*P*(Disease*X|*Symptom*A*, Symptom*B*, Symptom*C*)=*P*(Symptom*A|*Disease*X*)/*P*(Symptom*A*)*∗P*(Symptom*B|*Disease*X*)/*P*(Symptom*B*)*∗P*(Symptom*C|*Disease*X*)/*P*(Symptom*C*)*∗P*(Disease*X*)	73
	No prior probability	*P*(Disease*X|*Symptom*A*, Symptom*B*, Symptom*C*)/*P*(Disease*X*)=*P*(Symptom*A|*Disease*X*)/*P*(Symptom*A*)*∗P*(Symptom*B|*Disease*X*)/*P*(Symptom*B*)*∗P*(Symptom*C|*Disease*X*)/*P*(Symptom*C*)	67
	Log function form	log(*P*Disease*x*1*|*Symptom*A*, Symptom*B*, Symptom*C*)=log*P*(Symptom*A|*Disease*X*1)/*P*(Symptom*A|*Disease*X*2)+log*P*(Symptom*B|*Disease*X*1)/*P*(Symptom*B|*Disease*X*2)+log*P*(Symptom*C|*Disease*X*1)/*P*(Symptom*C|*Disease*X*2)+log*P*Disease*X*1/*P*Disease*X*2	76
	Equal probability	*P*(Disease*X|*Symptom*A*, Symptom*B*, SymptomC)=*P*(Symptom*A|*Disease*X*)/*P*(Symptom*A*)*∗P*(Symptom*B|*Disease*X*)/*P*(Symptom*B*)*∗P*(Symptom*C* *|*Disease*X*)/*P*(Symptom*C* )*∗P*(Disease*X*)	83

## Data Availability

The medical cases data used to support the findings of this study have not been made available because of patients' privacy.
